# Representations of lipid nanoparticles using large language models for transfection efficiency prediction

**DOI:** 10.1093/bioinformatics/btae342

**Published:** 2024-05-29

**Authors:** Saeed Moayedpour, Jonathan Broadbent, Saleh Riahi, Michael Bailey, Hoa V. Thu, Dimitar Dobchev, Akshay Balsubramani, Ricardo N.D. Santos, Lorenzo Kogler-Anele, Alejandro Corrochano-Navarro, Sizhen Li, Fernando U. Montoya, Vikram Agarwal, Ziv Bar-Joseph, Sven Jager

**Affiliations:** Digital R&D, Sanofi, Cambridge, MA, 02141, United States; Digital R&D, Sanofi, Toronto, ON, M5V 1V6, Canada; Digital R&D, Sanofi, Cambridge, MA, 02141, United States; Digital R&D, Sanofi, Cambridge, MA, 02141, United States; DataSentics, Brno 602 00, Czech Republic; mRNA Center of Excellence, Marcy L’Etoile, Sanofi, 69280, France; mRNA Center of Excellence, Sanofi, Waltham, MA, 02451, United States; mRNA Center of Excellence, Marcy L’Etoile, Sanofi, 69280, France; Digital R&D, Sanofi, Toronto, ON, M5V 1V6, Canada; Digital R&D, Sanofi, Cambridge, MA, 02141, United States; Digital R&D, Sanofi, Cambridge, MA, 02141, United States; mRNA Center of Excellence, Marcy L’Etoile, Sanofi, 69280, France; mRNA Center of Excellence, Sanofi, Waltham, MA, 02451, United States; Digital R&D, Sanofi, Cambridge, MA, 02141, United States; Digital R&D, Sanofi, Cambridge, MA, 02141, United States

## Abstract

**Motivation:**

Lipid nanoparticles (LNPs) are the most widely used vehicles for mRNA vaccine delivery. The structure of the lipids composing the LNPs can have a major impact on the effectiveness of the mRNA payload. Several properties should be optimized to improve delivery and expression including biodegradability, synthetic accessibility, and transfection efficiency.

**Results:**

To optimize LNPs, we developed and tested models that enable the virtual screening of LNPs with high transfection efficiency. Our best method uses the lipid Simplified Molecular-Input Line-Entry System (SMILES) as inputs to a large language model. Large language model-generated embeddings are then used by a downstream gradient-boosting classifier. As we show, our method can more accurately predict lipid properties, which could lead to higher efficiency and reduced experimental time and costs.

**Availability and implementation:**

Code and data links available at: https://github.com/Sanofi-Public/LipoBART.

## 1 Introduction

Lipid nanoparticles (LNPs) are a type of nanocarrier system used for the delivery of small molecules, siRNA drugs, and mRNA (Baden *et al.* 2021). Their prevalence and use in the drug industry greatly increased following the success of the mRNA COVID-19 vaccines (Hou *et al.* 2021). This has led to great interest in RNA-LNP delivery systems.

Naked RNA is a hydrophilic, negatively charged molecule that is naturally degraded in extracellular space by RNases. Therefore, the challenge in designing a complementary LNP is one that balances these properties and protects the mRNA molecule from degradation, yet promotes delivery to cells via endocytosis (Eygeris *et al.* 2021). This effect is achieved by combing a mixture of lipids. An ionizable lipid or cationic lipid is one key ingredient. It balances the negative charge and independently forms an encapsulated complex with the negatively charged RNA molecule (Hajj and Whitehead 2017). LNPs also destabilizes the endosome membrane and are neutral at physiological pH to minimize toxicity (Han *et al.* 2021, Sun and Lu 2023). Poly(ethylene glycol)-anchored lipids shield the surface of the nanoparticle from charge to reduce cytotoxicity (Meng *et al.* 2021). Helper lipids are added to promote the fusion of nanoparticles with cell membranes (i.e. 1,2-distearoyl-sn-glycero-3-phosphocholine; DSPC). Cholesterol fills in the gap between the other lipids for vesicle stability (Kim *et al.* 2021).

LNP success is defined by its transfection efficiency (TE), the ability of the LNP-mRNA complex to deliver the mRNA transcript to the target cell and induce the expression of the respective protein. TE is quantified by a proxy measurement of relative expression of a reporter gene such as luciferase, green fluorescent protein (GFP), or erythropoietin (Dara *et al.* 2019, Liu *et al.* 2021, Curtis *et al.* 2023).

LNP design can greatly benefit from a general optimization strategy. Machine learning (ML) methods showed great success in *de novo* drug design (Mouchlis *et al.* 2021). For instance, these methods can efficiently explore multidimensional parameter space of LNP components. Furthermore, ML methods can learn the complex relationship between the chemical entity and biological function of the LNPs. The availability and validity of the training data are the main bottleneck of developing ML methods for LNP property prediction. Ding *et al.* (2023) published one of the only publicly available LNP datasets where TE values for 622 LNPs along with the Simplified Molecular-Input Line-Entry System (SMILES) of the LNP components are provided. Moreover, they used this dataset to release a set of benchmarks for LNP TE prediction using a range of common classification models trained on a combination of physiochemical and geometric molecular fingerprints.

Other than the work of Ding *et al.* (2023), we are not aware of specific methods developed for LNP optimization. Several methods have been recently developed to optimize proteins and small molecules though these are not directly transferable to LNPs. For example, neural network architectures originally designed for natural language processing was extended to biomolecular systems, including mRNA and proteins, yielding valuable insights into the structure and function of large molecules (Lin *et al.* 2022, Li *et al.* 2023). In addition, previous works demonstrated that large language models (LLMs) based on SMILES strings can predict physiochemical and biological properties of small molecules. For instance, Winter *et al.* (2022) built a model based on the transformer architecture to predict activity coefficient phase equilibrium. Ross *et al.* (2022) designed a LLM based on SMILES that accurately predicts biomolecular properties and outperforms models trained using molecular geometry features in several downstream cheminformatics tasks. However, it has been observed that SMILES-based neural machine translation (NMT) models often generate invalid SMILES strings (Ucak *et al.* 2023).

Here, we exploited the power of both geometric and language-based embeddings to construct models for TE prediction of LNPs. We first tested a Graph Convolution Network (GCN) architecture for LNP optimization. Using the GCN architecture led to good performance on the benchmarks. Next, we extended these models by testing and developing several methods for embedding molecular features. Our methods were able to much more accurately model LNPs, leading to significant improvements over prior methods proposed for this task.

## 2 Materials and methods

### 2.1 Data

#### 2.1.1 Public benchmark dataset

We draw upon a benchmark dataset of LNPs with binary targets for satisfying and unsatisfying TE curated by Ding *et al.* (2023). The authors of this work have collected data from multiple published studies (Li *et al.* 2015, 2020, Zhang *et al.* 2020, Liu *et al.* 2021), which included measured *in vivo* protein expression following administration of a respective mRNA-LNP complexes.

The majority of these data are derived from Liu *et al.* (2021), who synthesized 572 LNPs using a novel class of multitailed ionizable phospholipids (iPhos). In lieu of cationic lipids, iPhos lipids are pH-switchable zwitterions and can form tissue-selective LNPs. To evaluate TE, they combined lipids with Fluc mRNA, administered the RNA-LNPs via injection, and measured luciferase expression [relative light unit (RLU) activity] 6 hours later with live animal imaging. Each LNP was assigned as either satisfying (*n* = 141) or unsatisfying or unsatisfying (*n* = 481) TE. ChemDraw molecule editor (CambridgeSoft) was used to build SMILES structures from the molecular structures described in figures in the associated papers.

#### 2.1.2 Multiclass benchmark dataset

The binary TE classes assigned across different studies in the public benchmark dataset described above used a variety of proxies for TE. For example, Liu *et al.* (2021) measured TE by expression of Fluc mRNA in the liver of mice, while in Li *et al.* (2020), TE is measured by luminescence of GFP following administration to HeLa cells. While both methods are valid for assessing TE, the assignment of satisfying TE across multiple studies becomes arbitrary without standardization of their relative control measurements.

Therefore, to improve the validity of our analysis, we curated a new dataset only using TE measurements from Liu *et al.* (2021). Here, TE measurements are discretely categorized into four classes of RLU activity (Supplementary Fig. S1), which also enables us to create multiclass classification targets, a more rigorous test for our set of prediction methods.

We used deep learning for chemical image recognition (DECIMER) (Rajan *et al.* 2020) to extract the SMILES strings from the structures of the lipid molecules displayed in the figures of the paper. The structures of the lipids were separated in two parts: dioxaphospholane oxides and amines. We followed the reaction rules described in the paper to connect the separate structures together. RLU readouts were extracted manually and set as a multiclass target for every SMILES string.

### 2.2 Embedding methods

An important step in quantitative structure-property relation (QSPR) is determining a suitable representation of the compound structures (i.e. lipids). To perform predictions, our classifiers require molecules to be represented by numerical vectors. We refer to this process as an embedding method because the molecule is embedded into a *d*-dimensional subspace. Ideally, the embedding provides the classifier with all of the structural information relevant to the downstream property prediction without including any unnecessary information (i.e. maximize signal-to-no ratio). Here, we explored five molecular embedding methods to determine which is most relevant for TE classification of LNPs.

#### 2.2.1 RDKit descriptors (expert)

The molecular descriptors provided by the RDKit library (https://www.rdkit.org/) were used to represent each lipid structure. These descriptors encompass a wide range of chemical properties, capturing structural and physicochemical characteristics of molecules. Leveraging domain expert, such features offer a nuanced and interpretable representation of molecular structures. Thus, each lipid can be represented as a 200-demensional vector (Open-source cheminformatics).

#### 2.2.2 Circular fingerprint

**Figure 1.**
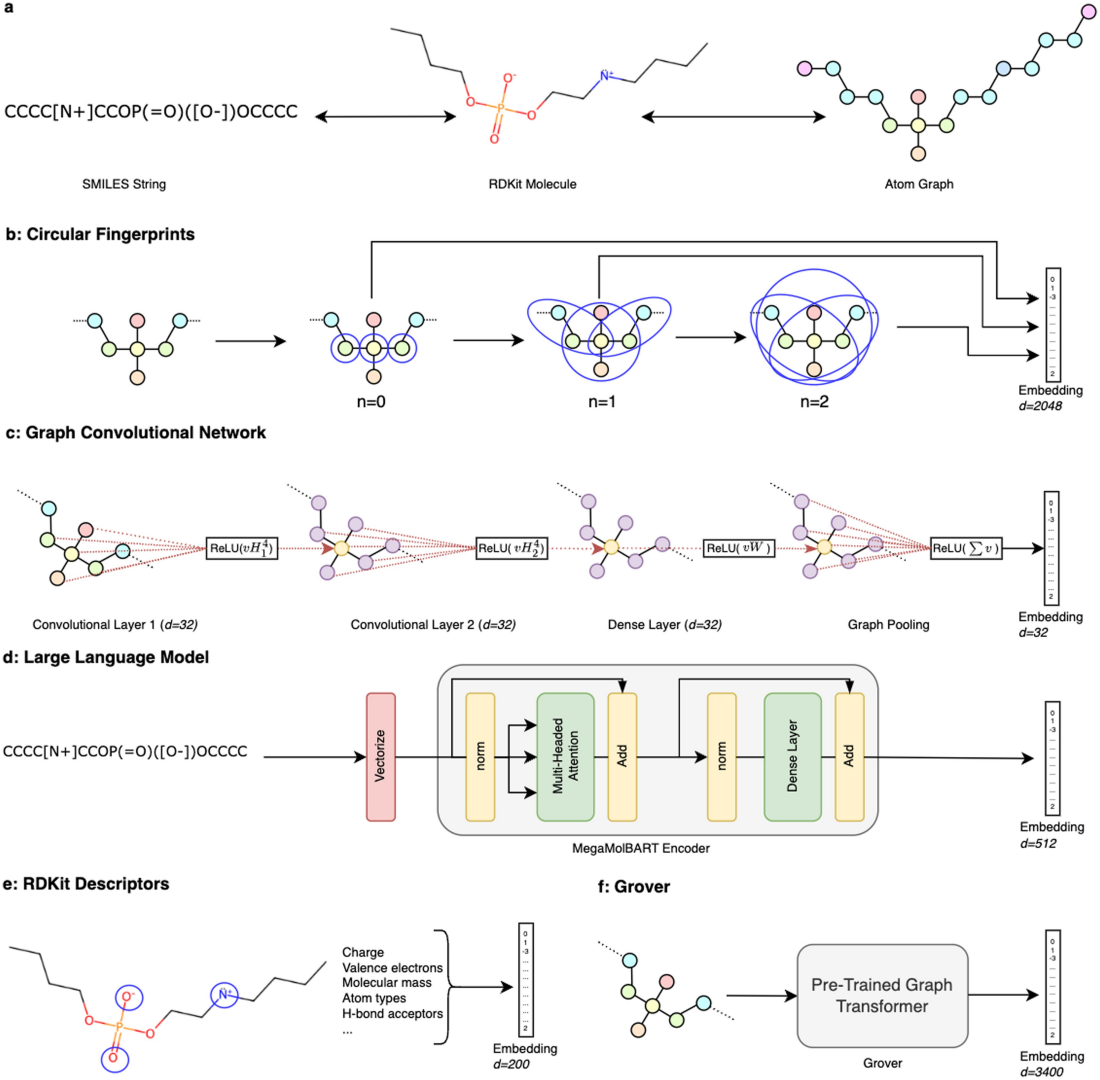
Embedding methods. (a) SMILES strings can be converted into atom graphs. Nodes are represented by 75 dimensional sparse vectors that encode their atom identity, number of valence electrons, and hydrogenation. (b) Circular fingerprints. At each step, circles of neighborhood size *n* are draw around every node. Distinct substructures are assigned a column in the embedding vector, and their relative count is accumulated. (c) Graph convolutional network (GCN). Hidden representations (purple/yellow) of every node in the graph are a function of pooling together information from respective neighboring nodes. Here, transformations are just shown for the center node (yellow). A final graph pooling step aggregates hidden representations from all nodes to output a single 32-dimensional embedding vector (Duvenaud *et al.* 2015). (d) Large language model. SMILES strings are first tokenized and embedded into an input vector. We use only the encoder portion of a pretrained MegaMolBART model (Irwin *et al.* 2022). (e) RDKit descriptors. Physiochemical and structural properties of the molecule are computed from the RDKit molecule and encoded into a 200 dimensional embedding. (f) Grover. We use a self-supervised graph transformer. This construct is similar to the graph convolutional network except a much larger model with multiclass attention, pretrained on small molecules (Rong *et al.* 2020).

Also referred to as Morgan fingerprints, circular fingerprints (CFPs) are formed by an iterative, deterministic algorithm. Briefly, substructures in the molecule are defined by drawing circles (max radius = 2) around every atom. Then, the number of different substructures is captured in a 2048-bit vector. In the current work, we use count-based CFPs which also captures the count of each substructure. The reason for this selection is that the lipids contain a lot of repeating substructures (Rogers and Hahn 2010) (Fig. 1b).

#### 2.2.3 Pretrained graph neural network (Grover)

A graph neural network lends itself well to molecule data as we can represent a molecule with a graph where atoms are nodes and bonds are edges. Grover is a self-supervised graph transformer trained on 11 million unlabeled molecules in the ZINC15 (Sterling and Irwin 2015) and ChEMBL (Gaulton *et al.* 2012) databases. We test embeddings both from Grover (GROVER_base_; ≈48M parameters; *d *=* *3400) and GroverLarge (GROVER_large_; ≈100M parameters; *d *=* *5000) (Rong *et al.* 2020).

#### 2.2.4 Customized graph convolution network (GCN)

The pretrained GCN model mentioned in Section 2.4.3 was not specifically tailored to lipids. We thus constructed a specific GCN to classify LNPs. We featurize our data by creating atom-based molecular graphs of the ionizable lipid structures. Here, the SMILES string of the ionizable lipid is converted to a graph where the nodes are atoms and the edges are bonds (Fig. 1a). It should be mentioned that in this representation, the structures are defined as hydrogen suppressed graphs.

Consider a phospholipid molecule, which is more complex than a simple fatty acid. The molecular structure can be abstracted as an undirected graph G=(V,E), where *V* is the set of vertices (atoms) and *E* is the set of edges (bonds) between them. To represent this molecule as an undirected graph, we consider each carbon (C), oxygen (O), phosphorus (P), and nitrogen (N) atom as vertices in the set *V*. Each bond between atoms is an edge in the set *E*. Thus, the hydrogen-suppressed graph for this phospholipid molecule can be abstracted as:


(1) $$G=(V,E),\;\text{where}\;V=C_{1},C_{2},\ldots ,C_{n},O_{1},O_{2},\ldots ,O_{m},P,N,$$

(2) $$\begin{array}{cc} & \begin{array}{cc} E= & (C_{i},C_{i+1}),(P,O_{l}),(O_{m},C_{p}),\\ & (N,C_{q}),|,1\leq i\leq n,1\leq l\leq m,p,q. \end{array}\\ & p\in \;\text{indices of carbon atoms in choline},\\ & q\in \;\text{indices of carbon atoms connected to}\;N \end{array}$$

Therefore, to capture the identity of each node such that the protonation states are distinguishable, we encode them with a feature vector of size *d *=* *75. This vector represents the element of each atom, valence electrons, and charge (Duvenaud *et al.* 2015).

The molecule graphs are then input into a GCN with two convolutional layers and a single dense layer (*d *=* *32). Each convolutional layer is composed of five different weight matrices (denoted $H_{l}^{i}$ for layer, *l* and 1≤i≤5), which correspond to the different number of neighbors a node can possibly have. These matrices are defined as:


(3) $$H_{i}^{l}=\text{ReLU}(D^{-\frac{1}{2}}\hat{A}D^{-\frac{1}{2}}H^{l-1}W_{i}^{l}),\;1\leq i\leq 5$$

Here, $H_{i}^{l}$ represents the output of the *i*-th convolutional filter at layer *l*, $\hat{A}$ is the adjacency matrix of the nodes with added self-connections, *D* is the degree matrix, $W_{i}^{l}$ is the weight matrix for the *i*-th convolutional filter at layer *l*, and ReLU is the activation function applied after the convolution.

At the final layer, node embeddings are aggregated together by simple summation to output a 32-dimensional representation of the whole molecule. Between each transformation, we apply a rectified linear unit (ReLU) function (Fig. 1c). We also apply a high dropout rate of 0.3 to prevent overfitting on our small dataset (Hinton *et al.* 2012).

Hyperparameters values are chosen in an internal validation process. For each set of hyperparameters, we performed k-fold cross-validation and scored the respective model by its average root mean squared error. We used Bayesian optimization to search a confined space of hyperparameters (Supplementary Fig. S5). Using these results, we chose a similar setup except with added regularization (high dropout rate in convolutional layers) and decreased complexity (less layers) to accommodate smaller dataset.

The GCN was trained for 5000 epochs, and we applied early-stopping with a patience of 500 epochs using cross-entropy loss on the validation set as our stopping metric. After training, we extract the embeddings and use them in the downstream classifier with the following loss function:


(4) $${\text{Loss}}_{\text{train}}=-\sum_{c=1}^{M}y_{o,c}\;\text{log}(p_{o,c})$$

where $y_{o,c}$ is the binary indicator if class label *c* is the correct classification for observation *o*, and $p_{o,c}$ is the predicted probability observation *o* is of class *c*. Early-stopping is based on validation loss, with a patience of 500 epochs.

#### 2.2.5 Fine-tuned large language model (MMB)

LLMs have shown recent success in understanding spoken languages. Similar architectures can be applied to the SMILES chemical language to provide information-rich embeddings of molecules. MolBART is such an LLM that combines a bidirectional encoder and autoregressive decoder similar to the original BART architecture (Lewis *et al.* 2019, Irwin *et al.* 2022). Here, we use MegaMolBART (MMB, https://docs.nvidia.com/bionemo-framework/0.4.0/models/megamolbart.html), which is the MolBART model pretrained on 1.45 billion molecules in the ZINC-15 database, using the NVIDIA Megatron framework (NVIDIA 2023). The majority of these molecules are not relevant to our lipid domain. To provide more relevant embeddings, fine tune the MegaMolBART base model (MMB) using 600 000 lipids from the SwissLipids database (https://www.swisslipids.org/) (Aimo *et al.* 2015). We segregated this data into 99% training, 0.5% validation, and 0.5% test splits. We trained over the whole dataset repetitively until the validation molecular accuracy plateaued at 18%. To generate embeddings of our lipid molecules, we input the SMILES strings into the encoder and collected the 512-dimensional embeddings from the model. We tested both the embeddings from the MMB and the MegaMolBART fine-tuned model (MMB-FT). Molecules are encoded into a string representation using notations like SMILES or SELFIES:


(5) $$S=\{s_{1},s_{2},\ldots ,s_{N}\}$$

where *N* is the sequence length, and each *s_i_* represents a token corresponding to an atom or bond in the molecule. The model comprises an encoder and a decoder, designed for bidirectional context understanding and autoregressive generation, respectively. The encoder processes the input sequence to produce contextualized representations:


(6) $$H=\text{Encoder}(S)=\{h_{1},h_{2},\ldots ,h_{N}\}$$

where **H** denotes the set of hidden states for each input token. The decoder generates the output sequence autoregressively:


(7) $$t_{i}=\text{Decoder}(t_{<i},H),\;\forall i\in \{1,\ldots ,M\}$$

aiming to predict each token *t_i_* based on previous tokens $t_{<i}$ and the encoded context **H**. The training objective is based on a denoising autoencoding strategy, aiming to reconstruct the original sequence from a corrupted version:


(8) $$L=-\sum_{i=1}^{N}\;\text{log}\;P(s_{i}|f(S_{\setminus i}),H)$$

where $f(S_{\setminus i})$ indicates the sequence with nod or removed tokens, and $P(s_{i}|\cdot )$ is the probability of the *i*-th token.

### 2.3 Classification of LNPs

In our analysis, we perform two classification tasks: binary classification and multiclass classification. In all of our methods, we opt to use the CatBoost classifier. The CatBoost is implemented as a machine learning library that specializes in gradient boosting for decision tree models. It is particularly well suited for small datasets and generally provides high-quality out-of-the-box results with minimal hyperparameter tuning. In the binary classification experiment, we applied a grid search for hyperparameters tuning and selected the model that performed the best on the validation dataset. In contrast with the binary classification task, our multiclass classification experiment relies on a smaller dataset; therefore, we opt to use a set configuration of the CatBoost classifier with a tree depth of 5, learning rate of 0.005, and 5000 iterations of training.

As mentioned in the introduction, LNPs contain four different lipid components. In the baseline classification method we compare to, they utilized embeddings of all four lipids and their relative proportions as input to their classification models (Ding *et al.* 2023). In our models, the input is solely the ionizable lipid embedding.

## 3 Results

We utilized a public benchmark dataset of LNPs for TE, sourced from Ding *et al.* (2023), where TE measurements were categorized into satisfying and unsatisfying categories. To improve analysis rigor, we curated a multiclass subset based solely on Liu *et al.*’s TE measurements, which discretize TE into four classes (Liu *et al.* 2021). Five molecular embedding methods, including RDKit Descriptors, Circular Fingerprint, Grover, Customized GCN, and Fine-Tuned Large Language Model (MMB), were explored for LNP classification. Our classification tasks involved binary and multiclass categorization, with the CatBoost classifier employed due to its suitability for small datasets and high-quality out-of-the-box performance. As a baseline, we replicated results from Ding *et al.* (2023), comparing the use of all four lipid embeddings versus only the ionizable lipid embedding in our classification model.

### 3.1 Evaluating method performance

We used two datasets to assess and compare the performance of our methods. The binary target dataset provided by Ding *et al.* (2023) and the multiclass target dataset, which we curated, based on the findings from Liu *et al.* (2021). As described in Section 2.1, the label in the binary dataset indicates if the level of TE is satisfying. In the multiclass dataset, the label represents the level of TE (RLU activity) categorized into four levels.

The binary classification experiments described in Ding *et al.* (2023) were reproduced using the same provided model hyperparameters and train, validation, and test data splits. Additionally, we trained CatBoost classification models using the five embeddings methods discussed: CFPs, Graph Convolutional Network (GCN), MegaMolBart base (MMB), and MegaMolBART-fine-tuned (MMB-FT) features that we generated by featurizing the SMILES strings in their dataset (Methods). We compared our methods to several other state-of-the-art chemoinformatic machine learning models (Gilmer *et al.* 2017, Goh *et al.* 2017).

Our results (Table 1) indicate that the CatBoost classifier performs generally well on this task with all embeddings. In particular, the CatBoost-MMB-FT and CatBoost-GCN architectures outperform all other methods, including those previously described by Ding *et al.* (2023).

**Table 1.** Classification benchmarks of the **public dataset**, curated by Ding *et al.* (2023), comprising 622 LNPs discretely separated into 141 LNPs with satisfying TE (labeled as 1) and 481 LNPs with unsatisfying TE (labeled as 0).

	Fingerprint					
Classifier	Expert	Grover	AUC	Balanced accuracy	F1 score	MCC
SVM	*✓*	x	0.961	0.906	0.848	0.795
SVM	x	*✓*	0.963	0.906	0.848	0.795
SVM	*✓*	*✓*	0.965	0.812	0.733	0.653
XGBoost	*✓*	x	0.947	0.843	0.774	0.701
XGBoost	x	*✓*	0.935	0.874	0.812	0.749
XGBoost	*✓*	*✓*	0.940	0.874	0.812	0.749
RF	*✓*	x	0.952	0.874	0.812	0.749
RF	x	*✓*	0.921	0.812	0.733	0.653
RF	*✓*	*✓*	0.922	0.832	0.750	0.665
MLP	*✓*	x	0.960	0.906	0.848	0.795
MLP	x	*✓*	0.938	0.916	0.875	0.832
MLP	*✓*	*✓*	0.683	0.854	0.800	0.741
CatBoost	*✓*	x	0.952	0.896	0.867	0.828
CatBoost	x	*✓*	0.959	0.874	0.812	0.749
CatBoost	*✓*	*✓*	0.960	0.874	0.812	0.749
Classifier	Embedding					
GCN	–		0.974	0.885	0.839	0.787
MPNN	–		0.964	0.874	0.813	0.749
ChemCeption	–		0.823	0.719	0.609	0.606
CatBoost	GCN		0.973	**0.916**	**0.875**	**0.832**
CatBoost	CFP		0.929	0.842	0.743	0.650
CatBoost	MMB		0.930	0.896	0.867	0.828
CatBoost	MMB-FT		**0.981**	0.875	0.857	0.831

Our methods are separated at the bottom of the table. AUC: area under receiving operating curve; MCC: Matthew’s correlation coefficient.

The highest area under the curve (AUC) results using the methods from Ding *et al.* (2023) was 0.965, whereas CatBoost-MMB-FT and CatBoost-GCN obtained slightly better results (AUC of 0.981 and 0.973, respectively). (Note that Ding *et al.* (2023) reported a slightly higher result in their work, likely resulting from different CV sets.) Since we are working with an imbalanced dataset, we also measured the balanced accuracy, F1 score, and Matthew’s correlation coefficient (MCC). We observe that the CatBoost-GCN outperforms all other models (balanced accuracy = 0.916; F1 = 0.875; MCC = 0.832 versus balanced accuracy = 0.906; F1 score = 0.848; MCC = 0.795).

We also tested several other classifiers for this problem (XGBoost, SVM, RF, MLP). We determined that CatBoost led to the best results and so only discuss CatBoost results here.

### 3.2 Multiclass cross-validated classification experiments

We used CatBoost for assessing the efficacy of different embeddings for the multiclass classification task. Here, we performed 5-fold cross-validation using five random folds stratified by class distribution. For each fold, we trained CatBoost models using identical hyperparameters and random seeds on all features and embeddings described in Section 2.2. The trained models were subsequently evaluated on the test fold. Due to imbalance in the dataset, several classification metrics that particularly consider class imbalance were employed to measure the performance, including area under the ROC curve using a one-versus-rest strategy (AUC), balanced accuracy, weighted average of F1 scores calculated for each class (weighted F1 score), and MCC.

The resulting performance metrics are provided in Table 2. The CatBoost model, trained with embeddings generated by our fine-tuned MegaMolBART model (MMB-FT), reached an average AUC, balanced accuracy, weighted F1 score, and MCC of 0.869, 0.573, 0.660, and 0.442, respectively. These results significantly improve upon results obtained using the best-performing embeddings from Ding *et al.* (2023) (Grover) for the AUC, balanced accuracy, and MCC. When trained with Grover embeddings, the CatBoost model resulted in an average AUC, balanced accuracy, weighted F1 score, and MCC of 0.839, 0.579, 0.643, and 0.411, respectively. To compare these results, we performed paired one-sided t-test statistics between the obtained classification metrics of all cross-validation folds for different embeddings in comparison to Grover embeddings (Supplementary Table S2). The MMB-FT embeddings lead to improvements over Grover embeddings with a *P* value of 0.034, 0.007, and 0.012 for AUC, weighted F1 score, and MCC, respectively. As shown in Fig. 3, there is a significant distribution shift in the obtained AUC, weighted F1 score, and MCC with MMB-FT embeddings in comparison to Grover embeddings. However, the Grover embeddings lead to higher balanced accuracy values.

**Table 2.** Cross-validated benchmarks of CatBoost multiclass classification model using different feature embeddings, based on the dataset derived from Liu *et al.* (2021).

Embds\Metrics	Cross-validation	AUC	Balanced accuracy	F1 score	MCC
MMB	Random	0.840	0.568	0.636	0.394
	Family holdout	–	0.377	**0.533**	**0.219**
MMB-FT	Random	**0.869**	0.573	**0.660**	**0.442**
	Family holdout	–	**0.379**	0.515	0.160
Expert	Random	0.831	0.566	0.644	0.410
	Family holdout	–	0.366	0.503	0.167
Grover	Random	0.839	**0.579**	0.643	0.411
	Family holdout	–	0.374	0.498	0.215
GroverLarge	Random	0.833	0.564	0.618	0.367
	Family holdout	–	0.363	0.492	0.120
CFP	Random	0.853	0.507	0.657	0.438
	Family holdout	–	0.374	0.518	0.162
GCN	Random	0.844	0.496	0.632	0.396
	Family holdout	–	0.371	0.515	0.155

Random refers to random 5-fold cross-validation with a fixed random seed, while Family holdout pertains to validation using the holdout lipid family group. The classification metrics represent mean values, averaged across all folds. The absence of AUC values for Family holdout validations is due to the mismatch in the number of test and predicted classes across multiple folds.

### 3.3 Generalizing to unseen holdout lipid families

Our multiclass classification dataset is drawn from Liu *et al.* (2021), who synthesized 572 LNPs and categorized them into families based on the number of hydrophobic tails and zwitterions of the ionizable lipid. Our observations revealed a significant correlation between these families and their respective TE (Supplementary Fig. S1a). Consequently, the random cross-validation described above may be influenced by the inclusion of certain LNP family members in the training set. To address this, we performed a more robust analysis where we held out LNP families. The LNPs were initially segregated into families. Subsequently, instead of splitting the set into random cross-validation folds, we designated one family as the test set and trained our model on the remainder of the dataset. This process was repeated for all seven LNP families in the dataset.

**Figure 2.**
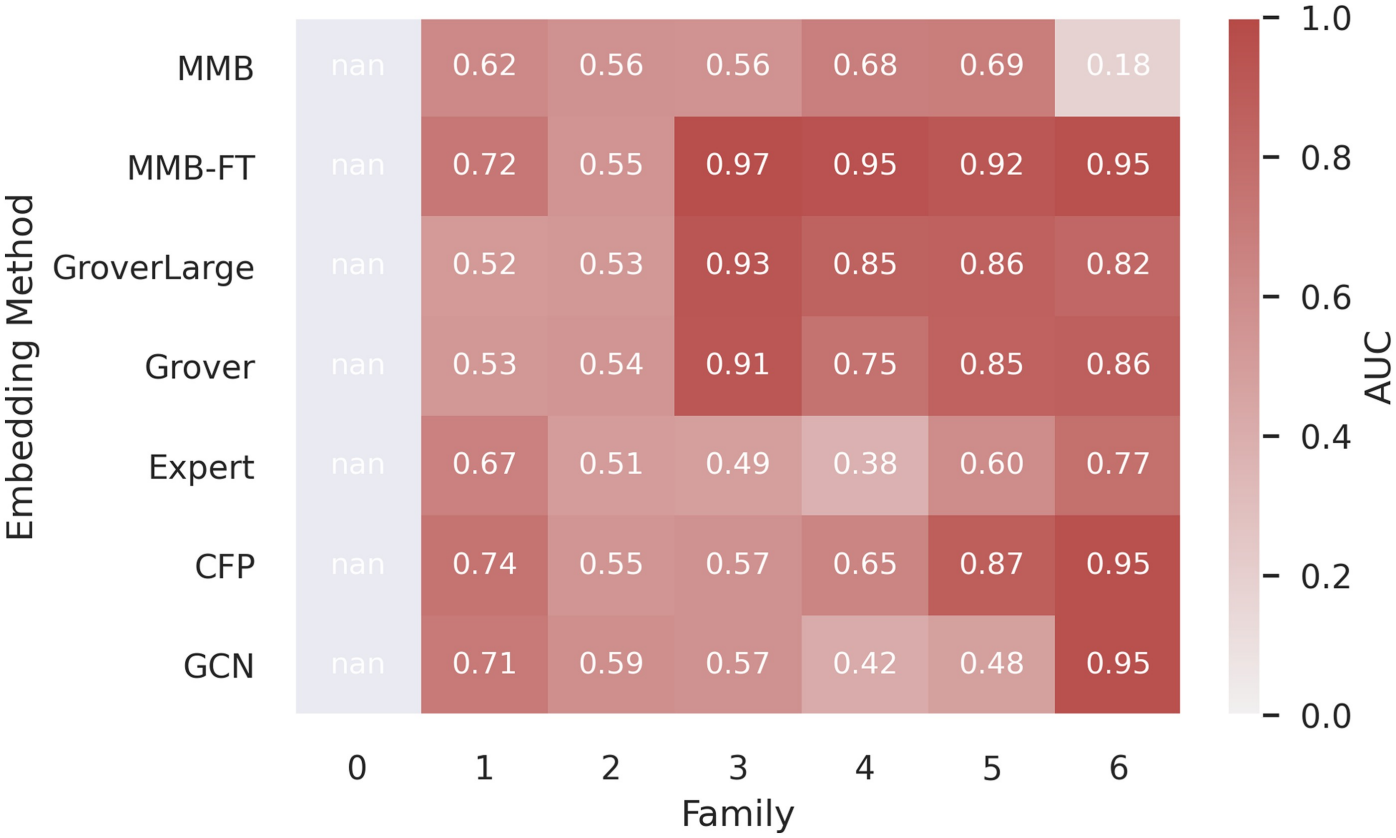
Comparison of embedding methods on binary classification of unsatisfying (RLU <10 000) versus satisfying TE (RLU ≥10 000) using a CatBoost classifier. Each column indicates the lipid family held out as the test set, classifier is trained on all other lipids.

Figure 2 shows the AUC performance of the binary classification task for trained CatBoost models using different embeddings. Family zero did not contain any LNPs with satisfactory TE; therefore, we excluded an evaluation of this family as a test set. We observed that MMB-FT embeddings outperform other embeddings and features. Specifically, the CatBoost-MMB-FT architecture generalizes well when families three to six are held out as the test set. The highest AUC metrics for families one to six were achieved with MMB-FT embeddings, with values of 0.72, 0.55, 0.97, 0.95, 0.92, and 0.95, respectively (while the Grover embeddings achieved 0.53, 0.54, 0.91, 0.75, 0.85, 0.86), indicating superior performance and generalizability for the binary classification task. In general, all methods struggle when family 1 and family 2 are held out as test sets. As shown in Table 2, the multiclass classification task demonstrated the best performance utilizing MMB and MMB-FT embeddings with average balanced accuracy, weighted F1 score, and MCC of 0.379, 0.660, and 0.219, respectively. See supplementary for a full analysis of the multiclass classification task.

**Figure 3.**
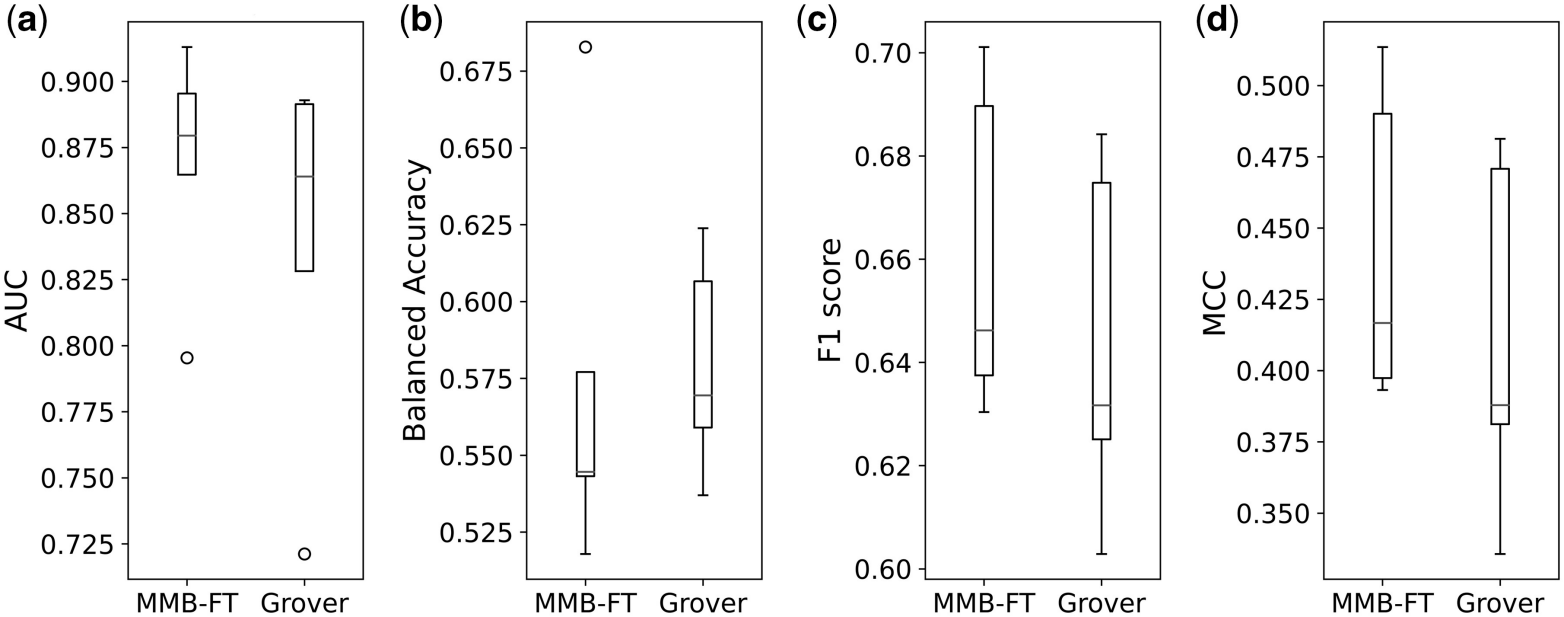
Visualization of performance metrics distribution shifts for the 5-fold cross-validated multiclass classification experiments with MMB-FT and Grover embeddings. The box plots present (a) AUC, (b) balanced accuracy, (c) weighted F1 score, and (d) Matthew’s correlation coefficient metrics.

### 3.4 Clustering the embeddings

To compare the embedding spaces of our different embedding methods, we used Uniform Manifold Approximation and Projection (UMAP) to visualize them in two dimensions. The projections were color-coded based on the LNP family (Supplementary Fig. S3) and the LNP TE class (Supplementary Fig. S4). We observed that both MMB and MMB-FT embeddings exhibited clusters that correlated well with their respective lipid family class. However, none of the employed embedding methods yielded clusters that aligned with their respective TE class.

We further quantified our observation by performing *K*-means clustering on the 572 iPhos lipids using each embedding method. Supplementary Table S1 presents the computed normalized mutual information scores between the identified clusters and those defined by their respective family and TE classes. Notably, MMB-FT embeddings exhibited a high mutual information score of 0.626 with the LNP family class. In contrast, expert embeddings have a mutual information score of 0.596.

## 4 Discussion

Lipids play a pivotal role in mRNA vaccines, serving as crucial components that encapsulate and protect the fragile molecules. LNPs not only ensure the stability and integrity of the mRNA but also facilitate its efficient delivery into cells (Kon *et al.* 2022). The representation of lipid structures into numerical vectors poses a significant challenge due to the limited availability of public structure-activity data. To improve models for LNP design and optimization, we have used and extended five different molecular representation methods for predicting the TE of LNPs.

Although our models are based on the dataset provided by Ding *et al.* (2023), our approach differs in several key aspects. First, instead of using all four components of the LNPs, we only used the embedding of the ionizable lipid to construct our models. This puts the focus of our model on the lipid molecules and eliminated uncertainties in other LNP factors such as the component ratio. Moreover, we extracted a subset of the dataset pertaining to pH-switchable zwitterions (Liu *et al.* 2021) and created a multiclass classification dataset, with four distinct classes of LNPs based on their TE values. This provides a more challenging task for LNP classification. Furthermore, in our work, we evaluate a more extensive range of embedding methods and provide an analysis on the most optimal way to numerically represent lipid molecules for TE classification.

The methods we tested include RDKit descriptors, CFPs, a pretrained graph transformer (Grover), in addition to our GCN architecture and a fine-tuned MegaMolBART model. We employed the described methods to generate molecular embeddings for lipid structures in two datasets: a public dataset published by Ding *et al.* (2023) for a binary target of TE and a multiclass TE target subset we curated based on the work published by Liu *et al.* (2021).

We observed that the top-performing classification method was predominantly CatBoost-MMB-FT. This method showed superior performance in the binary classification task with an AUC of 0.981. Additionally, it demonstrated significant improvement for the multiclass classification task. Notably, the CatBoost-MMB-FT method was the only method showing statistically significant improved performance compared to Grover embeddings.

Our embedding methods can be grouped based on the initial featurization of their originating molecule, as illustrated in Fig. 1. Three of these methods, namely, Grover, CFP, GCN, are constructed from atom graphs, another one employs the RDKit molecule object, and the LLM generates embeddings from SMILES strings. Since it uses the minimal amount of structural data, the text LLM method exhibits the least restrictions. Unquestionably, atom connectivity contributes vital information to the prediction task. Nevertheless, multiple aspects of intra- and intermolecular forces’ connectivity, which significantly influence LNP formation beyond covalent bonds, are essential (Liu *et al.* 2021). Multiple SMILES strings can result in the same structure. We hypothesize here that due to the relaxation of structure in the SMILES string, the MegaMolBART model can learn to consider patterns in the molecule beyond bonding, which may, in fact, be more crucial to LNP formation and efficacy. Unlike small molecules, LNPs are formed from thousands of repeating units put together, so intermolecular forces become much more important in this domain (Kauffman *et al.* 2015, Meng *et al.* 2021, Kon *et al.* 2022).

Unsupervised fine-tuning of MMB on the Swiss Lipids dataset enabled effective generalization when the method was later applied to a small labeled dataset. This highlights a novel aspect of our methodology in contrast to prior methods.

Among all the methods presented in this paper, we anticipate that those based on neural networks will exhibit substantial performance scalability with an increment in training data. The domain of LNP formulation is an emerging field. Thus, while the available data are limited, the high potential of mRNA vaccines and drugs will likely lead to a surge in publicly available LNP datasets. Both the data that we used for this paper and the methods we developed are available on the GitHub page.

## Supplementary Material

btae342_Supplementary_Data
