# Supplementary material for: Representations of lipid nanoparticles using large language models for transfection efficiency prediction
Source: Bioinformatics. 2024 May 29;40(7):btae342. doi: 10.1093/bioinformatics/btae342 (PMC11629694; doi:10.1093/bioinformatics/btae342)
Supplement: btae342_Supplementary_Data [file btae342_supplementary_data.pdf]

Supporting Methods and Results for: Representations of Lipid Nanoparticles Using Large Language Models for Transfection Efficiency Prediction

Saeed Moayedpour[1,†], Jonathan Broadbent[2,†], Saleh Riahi[1], Michael Bailey[1], Hoa Vu Thu[5], Dimitar Dobchev[4], Akshay Balsubramani[3], Ricardo Nascimento Dos Santos[4], Lorenzo Kogler-Anele[2], Alejandro Corrochano-Navarro[1], Sizhen Li[1], Fernando Ulloa Montoya[4], Vikram Agarwal[3], Ziv Bar-Joseph[1,*], and Sven Jager[1,*]

[1]Digital R&D, Sanofi, Cambridge, MA, USA
[2]Digital R&D, Sanofi, Toronto, Ontario, Canada
[3]mRNA Center of Excellence, Sanofi, Waltham, MA, USA
[4]mRNA Center of Excellence, Sanofi, Marcy L'Etoile, France
[5]DataSentics, Brno, Czech Republic

May 20, 2024

1 Supporting Methods and Results

Due to the difference in the number of predict and test classes across multiple folds in the unseen holdout setup, AUC could not be calculated. We observed that multi-class classification with held out families as a test set, presents a significant challenge for all of our models, with no substantial difference noted in performance scores (Figure S2a). For this task, a strong correlation was only observed between multi-class accuracy and binary classification AUC from the large self-supervised models including Grover, GroverLarge, and MMB-FT (Figure S2b).

	**Mutual Information**	
**Embedding Method**	Family	TE
CFP	0.356	0.096
Expert	0.596	0.137
Grover	0.406	0.123
Grover large	0.400	0.127
GCN	0.017	0.003
MegaMolBART base	0.578	0.122
MegaMolBART finetuned	0.626	0.133

Table S1: K-means clustering of 572 iPhos lipids [1], conducted using various embedding methods. The table presents the computed normalized mutual information scores between the clusters and two classes: (a) lipid family class, and (b) TE class.

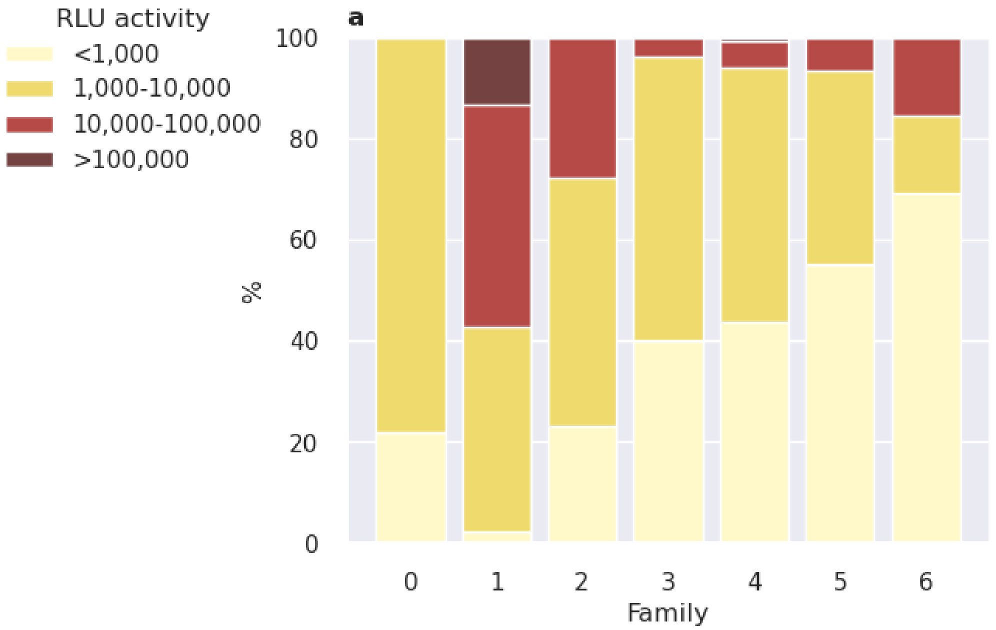

	Family 0	Family 1	Family 2	Family 3	Family 4	Family 5	Family 6
Total Number of lipids	78	91	65	130	117	78	13
Number of tails	1	1	1	2	3	4	5
Number of zwitterions	2	3	4	2	3	4	5

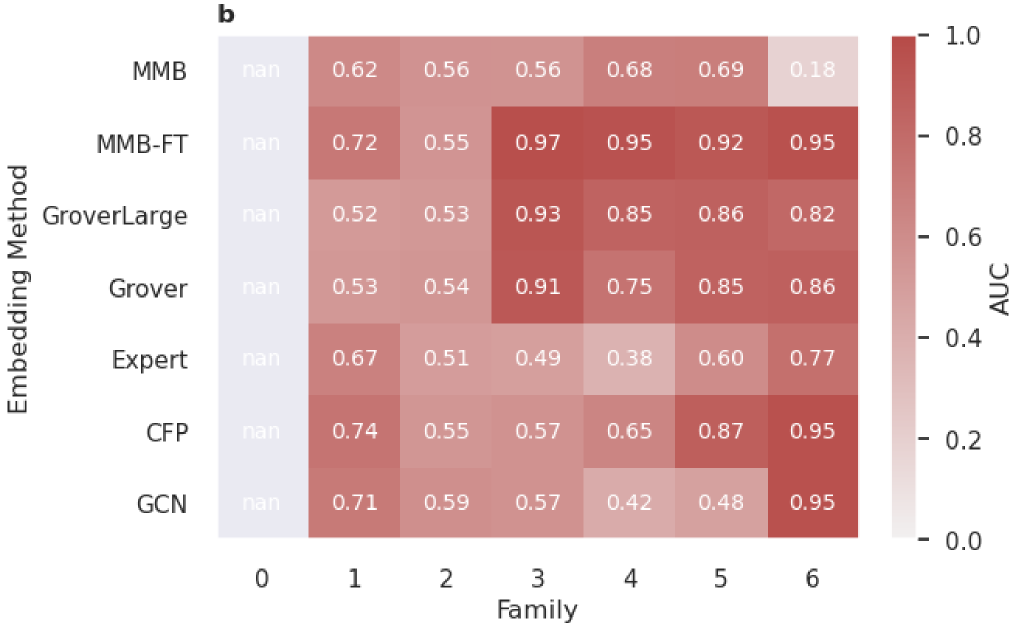

Figure S1: We demonstrate how each embedding method generalizes to unseen lipid families. **(a)** Tranfection Efficiency (RLU) distribution of 572 iPhos LNPs segregated into families based on count of hydrophobic tails and zwitterions. **(b)** Comparison of embedding methods on binary classification of unsatisfying (RLU $< 10,000$) vs satisfying TE (RLU $\geq 10,000$) using a CatBoost classifier. Each column indicates the lipid family held out as the test set, classifier is trained on all other lipids.

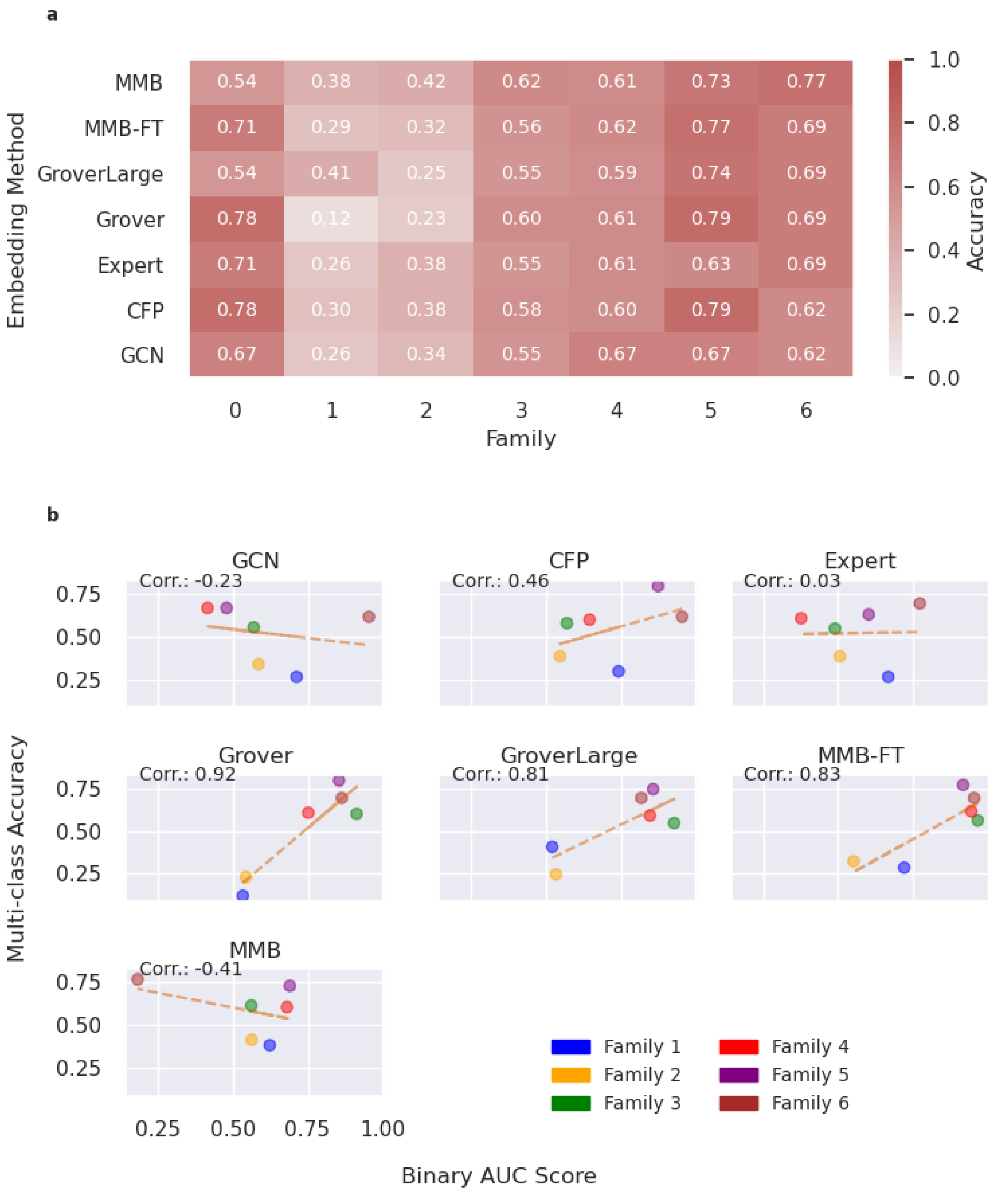

Figure S2: **(a)** Similar experiment to figure S1b with multi-class classification accuracy as the evaluation metric. **(b)** Correlation of binary classification and multi-class classification scores [1]

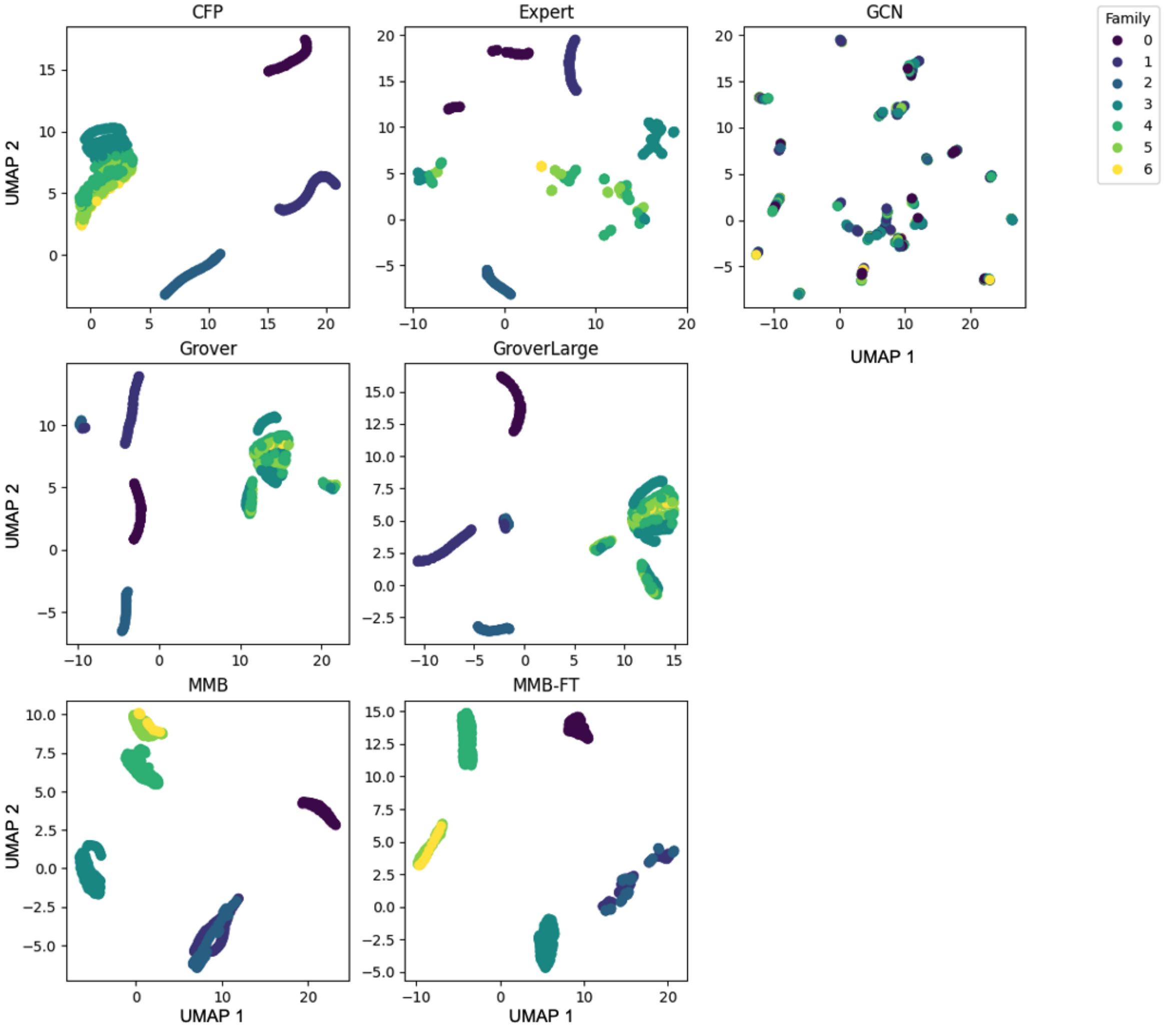

Figure S3: UMAP clustering of lipid families using different embeddings: a) CFP b) Expert c) GCN d) Grover e) GroverLarge f) MMB g) MMB-FT

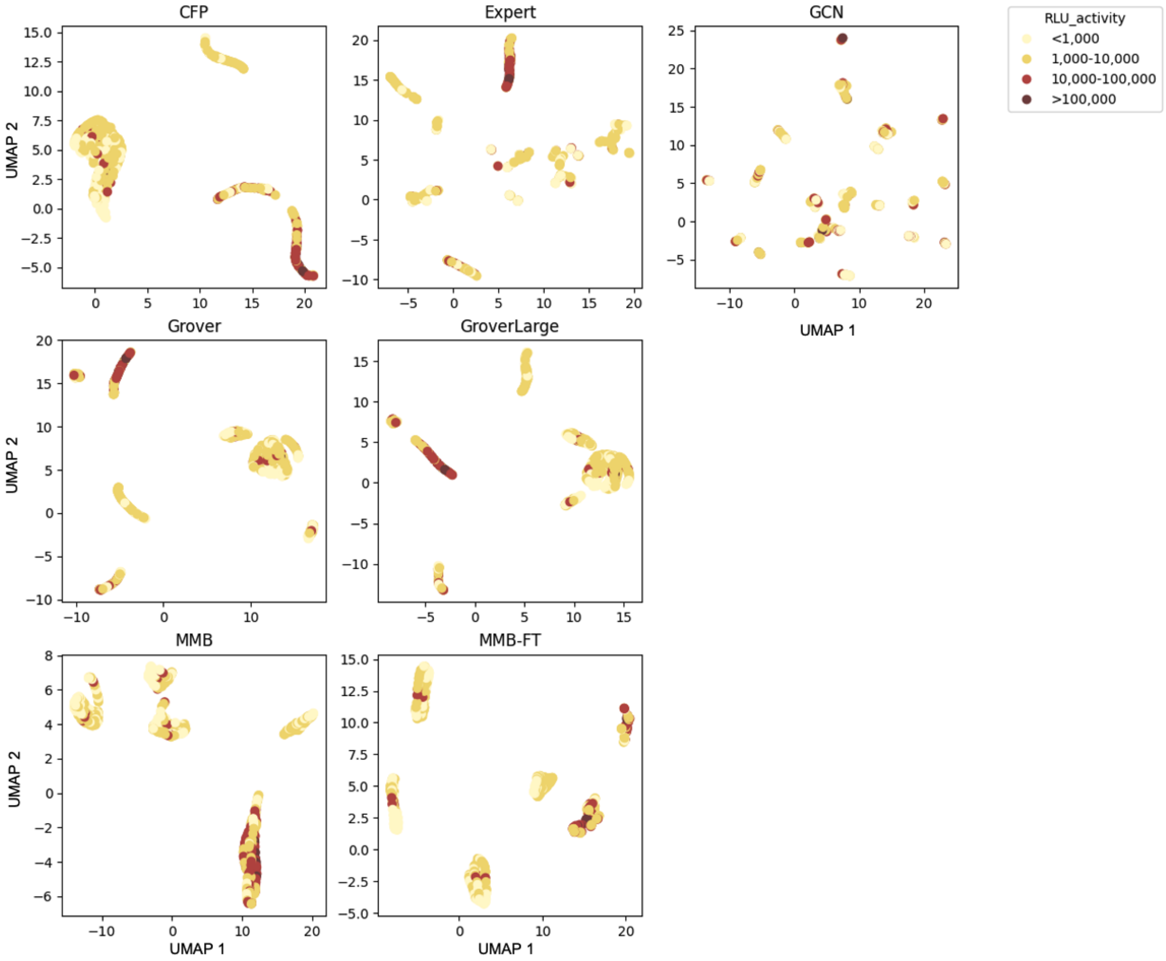

Figure S4: UMAP clustering of lipid RLU classes using different embeddings: a) CFP b) Expert c) GCN d) Grover e) GroverLarge f) MMB g) MMB-FT

Embeddings	Metrics	T-stat	P-value
MegaMolBART base	AUC	0.090	0.466
	Balanced Accuracy	-0.285	0.605
	F1-score	-0.529	0.687
	MCC	-0.638	0.721
MegaMolBART fine tuned	AUC	2.462	**0.034**
	Balanced Accuracy	-0.165	0.562
	F1-score	4.085	**0.007**
	MCC	3.492	**0.0125**
CFP	AUC	0.991	0.188
	Balanced Accuracy	-3.02	0.980
	F1-score	1.311	0.129
	MCC	1.33	0.127
Expert	AUC	-3.516	0.987
	Balanced Accuracy	-0.653	0.725
	F1-score	1.311	0.129
	MCC	-0.047	0.517
GCN	AUC	0.531	0.311
	Balanced Accuracy	-1.664	0.914
	F1-score	-0.864	0.782
	MCC	-0.754	0.754

Table S2: Comparative analysis of the paired t-test between cross-validated multi-class classification metrics of the CatBoost model using different embeddings, relative to grover embeddings based on the greater mean hypothesis.

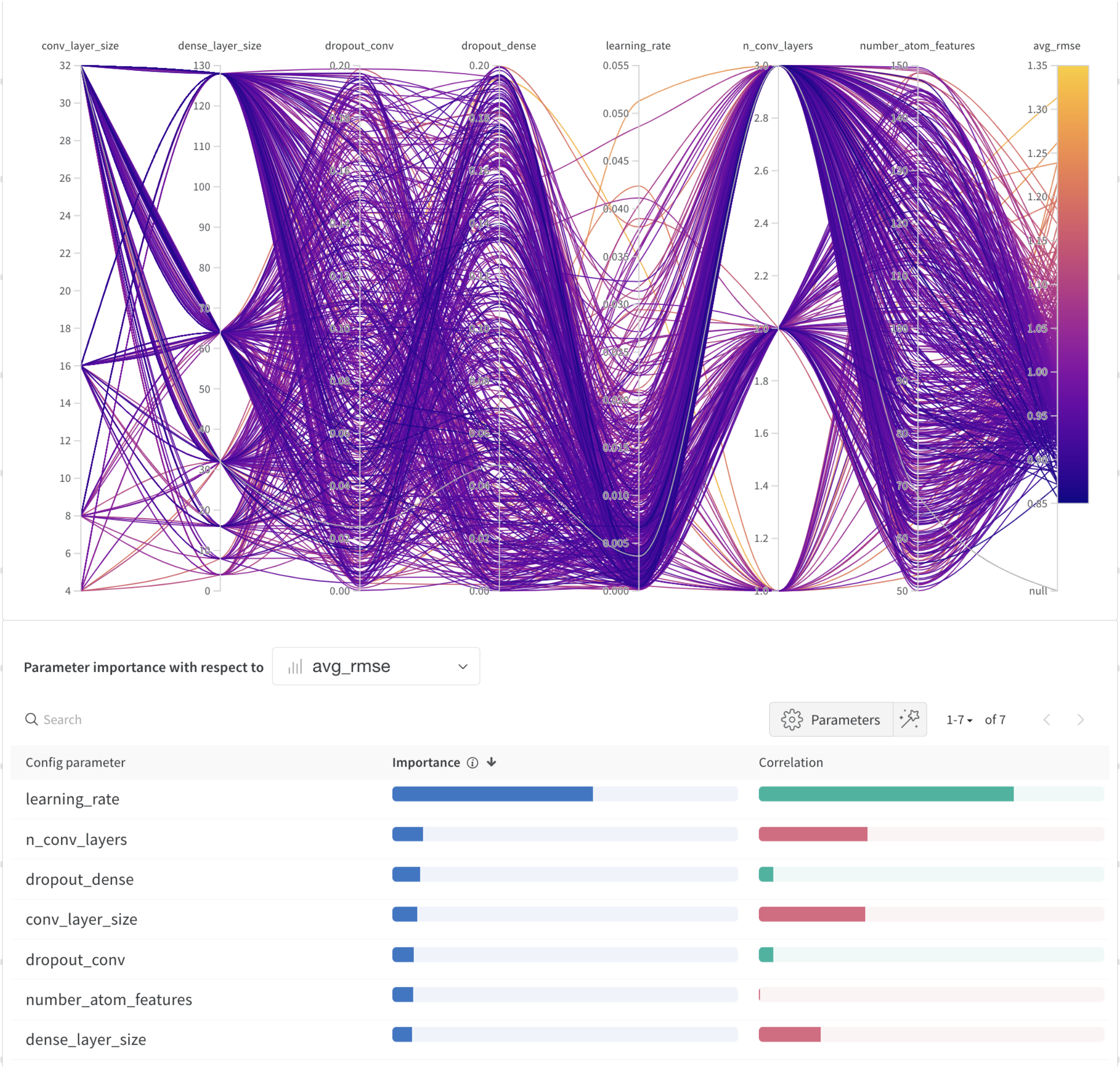

Figure S5: We performed a sweep for hyperparameters of our GCN using Weights and Biases (wandb). Each line represents a train and evaluation run with 5-fold cross-validation. We trained and evaluated the model on our **internal dataset**, using root mean squared error as our target metric. Using these results we chose similar hyperparameters with added regularization to account for the small dataset.

References

[1] Shuai Liu et al. "Membrane-destabilizing ionizable phospholipids for organ-selective mRNA delivery and CRISPR–Cas gene editing". In: *Nature materials* 20.5 (2021), pp. 701–710.
